# Temporal trends of morbidities, and risk and protective factors for noncommunicable diseases in elderly residents in Brazilian capitals

**DOI:** 10.1590/1980-549720230009.supl.1

**Published:** 2023-04-21

**Authors:** Alanna Gomes da Silva, Fabiana Martins Dias de Andrade, Edmar Geraldo Ribeiro, Deborah Carvalho Malta

**Affiliations:** I Universidade Federal de Minas Gerais, School of Nursing, Graduate Program in Nursing – Belo Horizonte (MG), Brazil.; II Universidade Federal de Minas Gerais, School of Medicine, Graduate Program in Public Health – Belo Horizonte (MG), Brazil.

**Keywords:** Health of the elderly, Noncommunicable diseases, Risk factors, Protective factors

## Abstract

**Objective:**

To analyze the temporal trends of prevalence of morbidities, risk and protection factors for noncommunicable diseases in elderly residents in Brazilian capitals between 2006 and 2021.

**Methods:**

A time series study with data from the Surveillance System of Risk and Protective Factors for Chronic Diseases by Telephone Inquiry. The variables analyzed were: high blood pressure, diabetes, smoking, overweight, obesity, consumption of alcoholic beverages, soft drinks, fruits and vegetables, and the practice of physical activity. Prais-Winsten regression and Interrupted Time Series from 2006 to 2014 and 2015 to 2021 were used.

**Results:**

From 2006 to 2021, for the total elderly population, there was an increase in diabetes (19.2 to 28.4%), alcohol consumption (2.5 to 3.2%), overweight (52.4 to 60.7%) and obesity (16.8 to 21.8%), and a reduction in the prevalence of smokers (9.4 to 7.4%) and in soft drink consumption (17 to 8.7%). By the interrupted series, between 2015 and 2021, there was stability in the prevalence of diabetes, female smokers, overweight among men, obesity in the total and male population, and soft drink consumption.

**Conclusion:**

Over the years, there have been changes and worsening in the indicators analyzed, such as an increase in diabetes, alcohol consumption, overweight, and obesity, which reinforces the importance of continuous monitoring and sustainability programs to promote the health, especially in the context of economic crisis, austerity, and COVID-19 pandemic.

## INTRODUCTION

Brazil is in a process of demographic and epidemiological transition due to the decrease in fertility and birth rates, as well as the increase in life expectancy, the reduction of infectious diseases and the growth of noncommunicable chronic diseases (NCDs).^1^.

Population aging is considered one of society's main gains, as it reflects social, technological, and health advances^2^. The United Nations Organization estimated that the global population aged 65 years old or older is expected to increase from 10% in 2022 to 16% in 2050^3^. In Brazil, projections by the Brazilian Institute of Geography and Statistics (*Instituto Brasileiro de Geografia e Estatística* – IBGE) show that the aged population will correspond to 18.6% in 2030 and 33.7% in 2060^4^.

NCDs are the most frequent among the aged population and the main causes of death and disability^5^. The four main groups of NCDs (cardiovascular, cancer, chronic respiratory diseasesand diabetes) have modifiable and common risk factors, such as smoking, alcohol abuse, physical inactivity and inadequate diet, which entail numerous social and economic consequences for individuals, families, and society^6^. On the other hand, the adoption of healthy habits, such as the practice of physical activity and the consumption of fruits and vegetables, can reduce the risk of developing these diseases, in addition to helping to control weight and improve quality of life and mental health^7^.

Due to the severity of NCDs and their impact on Public Health, national efforts have been made to implement policies, programs and actions for the prevention and control of these diseases and their risk factors, in addition to comprehensive care and treatment^6,7^. There are also policies and guidelines that propose actions, services, and programs aimed at comprehensive health care for the aged^8,9^. However, from mid-2014, economic and governmental crises occurred in Brazil, with the implementation of austerity policies that contributed to generate unemployment, increase inequalities, and negatively affect the health and social protection system^10,11^. Added to this is the COVID-19 pandemic, which contributed to reduced access to health services and worsened living conditions, especially among aged people with NCDs^12,13^.

In the national context of population aging and increased NCDs cases, accompanied by the crisis, austerity and COVID-19 pandemic scenario, few Brazilian studies have been carried out, especially with analyses of historical series. In addition, it is important to monitor the health indicators of the aged population, in order to verify the changes that have occurred over the years and to know the reality in which they are inserted and their life habits. Thus, it is expected to contribute to the surveillance, control, and prevention of NCDs, so that Brazilian aged people can achieve longevity with quality of life.

This article aimed to analyze the temporal trends in the prevalence of morbidities and risk and protective factors for NCDs in elderly residents in Brazilian capitals between 2006 and 2021.

## METHODS

### Study design and data source

This is a time series study, from 2006 to 2021, with data from the Surveillance System of Risk and Protective Factors for Chronic Diseases by Telephone Survey (*Sistema de Vigilância de Fatores de Risco e Proteção para Doenças Crônicas por Inquérito Telefônico* – Vigitel).

Vigitel is a population-based telephone survey that annually monitors, since 2006, the frequency and distribution of the main risk and protective factors for NCDs^14^. The sampling procedures employed aim to obtain representativeness of the capitals of the 26 Brazilian states and the Federal District, through probabilistic samples of the population of adults (≥18 years old) residing in households with at least one telephone landline^14,15^.

### Sample

This study considered the population aged 65 years old or older, as it is the same used for post-stratification in the Vigitel analyses.

Between 2006 and 2022, approximately 162,673 people aged 65 years old or older were interviewed, as follows: 6,069 (2006); 6,215 (2007); 6,679 (2008); 6,886; (2009); 7,340 (2010); 8,074 (2011); 7,580 (2012); 10,400 (2013); 8,417 (2014); 13,349 (2015); 13,649 (2016); 15,727 (2017); 15,338 (2018); 17,583 (2019); 8,941 (2020); and 10,426 (2021).

### Variables

For the present study, the following variables evaluated by Vigitel were included:

- Morbidity: high blood pressure and self-reported diabetes.
- Risk factors: smoking; abusive consumption of alcoholic beverages; overweight; obesity; and soft drink consumption (variable added to Vigitel in 2007).
- Protective factors: recommended consumption of fruits and vegetables (variable added to Vigitel in 2008) and practice of leisure-time physical activity (LTPA) (variable added to Vigitel in 2009).

The full description of the variables is in Supplementary Material 1.

### Data analysis

The variables were described in terms of prevalence by year the survey was carried out and stratified according to the total aged population and sex (female and male).

This study used the analysis of interrupted time series, a methodology that aims to verify whether there was an effect of intervention/event/event on the trends of a given measure of interest^16^. Thus, the following periods and the respective events were considered for the analysis: 2006 to 2021 (complete period); 2006 to 2014 (implementation of the strategic action plan to face NCDs in Brazil 2011–2022 and economic stability)^6,11^; and 2015 to 2021 (occurrence of economic crisis, with implementation of austerity policies^17,18^, and COVID-19 pandemic, recognized by the World Health Organization [WHO], on March 11^th^, 2020)^19^.

Trend analysis was performed using generalized Prais-Winsten linear regression, which corrects for the effect of first-order serial autocorrelation^20^.

The annual percentage change (APC) was also calculated for each analyzed variable, based on the following formula^21^:


APC=(−1+10^β1)×100%

Beta 1 (β1) refers to the angular coefficient of the Prais-Winsten regression.

The 95% confidence intervals (95%CI) of the APC measurements were also calculated, using the following formula^21^:


Minimum95%CI=(−1+10^[β1−t×e])×100%

Maximum95%CI=(−1+10^[β1+t×e])×100%

Where: the *t* in the formula refers to the Student's *t* test by the degrees of freedom for time periods and with a confidence level of 95%, while *e* corresponds to the standard error. Prais-Winsten regression and standard error β1 values were generated using a statistical analysis program.

Regression results were interpreted as follows: significant trend when the β of the regression was different from zero and the p-value was lower than or equal to 0.05. Thus, there was an increasing trend when β was positive, a decreasing trend if β was negative, and a stationary trend when no statistically significant difference was identified.

For data analysis, post-stratification weights used in Vigitel were considered^14,15^.

Analyses were performed using Stata software (Stata Corp. LP, College Station, Texas, United States), version 14.0, and data organization and graphing were performed using Microsoft^®^ Office Excel 2016.

### Ethical aspects

Vigitel complied with Resolution 466/2012, which involves research with human beings. The data are available for public access and use, and their collection was approved by the National Commission for Ethics in Research on Human Beings of the Ministry of Health. The informed consent was obtained orally at the time of telephone contact with the interviewees.

## RESULTS

**Figure 1:**
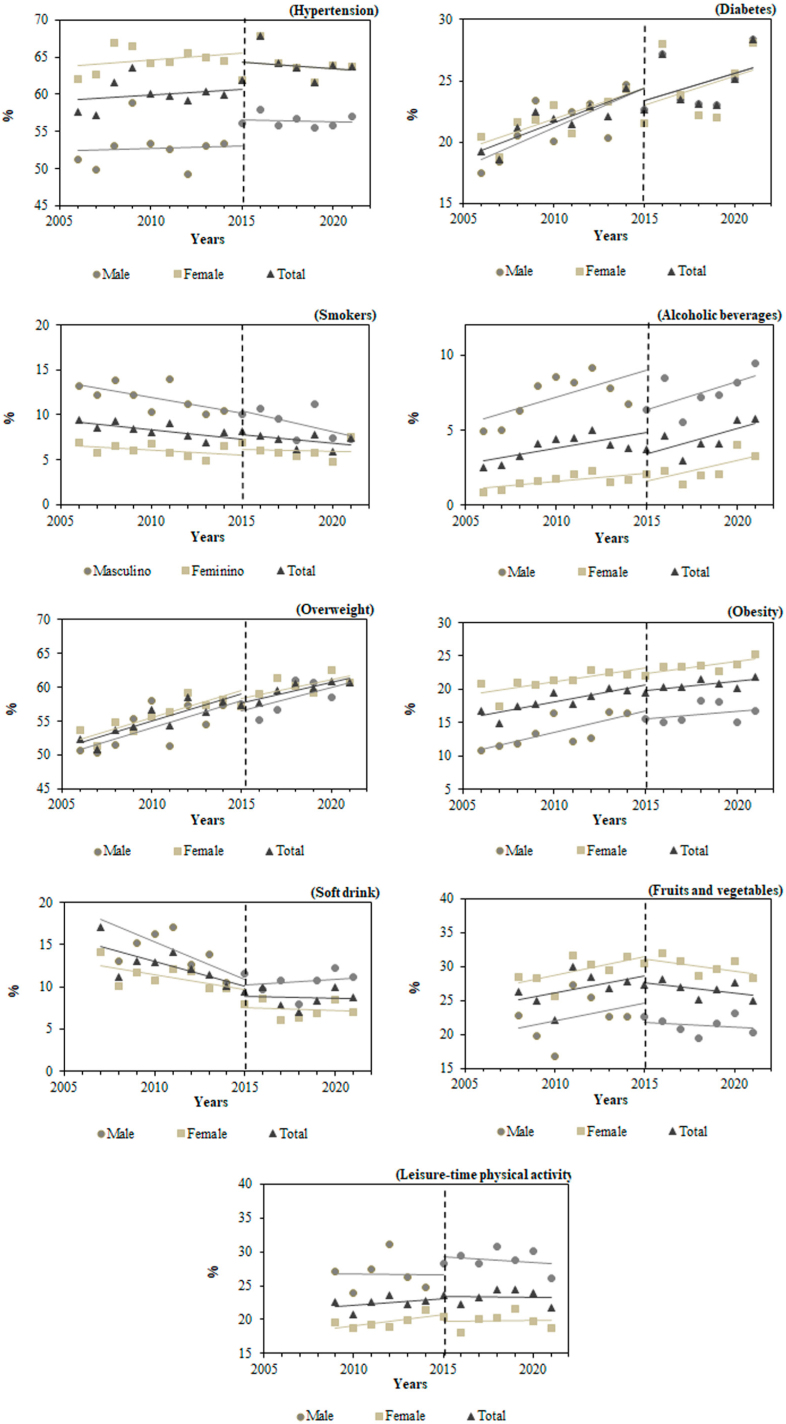
Interrupted time series of the prevalence of high blood pressure, diabetes, risk and protective factors for noncommunicable chronic diseases. Vigitel. Brazilian capitals from 2006 to 2021.

Figure 1 represents the time series of the prevalence of high blood pressure and diabetes, as well as the risk and protection factors for NCDs, from 2006 to 2021, according to sex, with the representation of interruption, which makes it possible to visualize variations in the prevalence of indicators from 2015. Full results can be found in Supplementary Material 2.

Self-reported high blood pressure increased significantly among aged males (APC=0.66%; 95%CI 0.06–1.27). Diabetes showed a growing trend for the total aged population (APC=1.88; 95%CI 1.13–2.64), males (APC=2.32; 95%CI 1.56–3.10), and females (APC =1.60; 95%CI 0.77–2.44) (Figure 1).

With regard to risk factors, considering the entire period, the trend was for a decline in the prevalence of smoking among all aged people (APC=−2.16; 95%CI −2.77; −1.54) and for those of the male sex (APC=-3.50; 95%CI −4.38; −2.62). On the other hand, among females, the trend was one of stability. Alcohol abuse showed a growing trend among the total aged population (APC=3.16; 95%CI −0.09; 6.51) and females (APC=6.33; 95%CI 2.65–10.15), with stability among males. As for overweight, there was an upward trend for the total number of aged people (APC=1.11; 95%CI 0.92–1.30), among males (APC=1.13; 95%CI 0.77–1.50) and among females (APC=1.07; 95%CI 0.85–1.29). Similarly, obesity also increased significantly, respectively: APC=1.87 (95%CI 1.29–2.45), APC=2.84 (95%CI 1.51–4.18), and APC= 1.47 (95%CI 1.03–1.92). For soft drinks consumption, the trend was decreasing for the total aged population (APC=−4.32; 95%CI −6.25; −2.35), among male participants (APC=-3.99; 95%CI −6.08; −1.86), and among females (APC=−4.73; 95%CI −6.74; −2.68) (Figure 1).

As for the protective factors for NCDs, the consumption of fruits and vegetables and the practice of LTPA remained stable for the total population and for both sexes (Figure 1).

Tables 1 and 2 show the results of the interrupted time series, carried out in two periods (2006 to 2014 and 2015 to 2021), respectively, according to sex.

**Table 1:** Interrupted time series of the prevalence of high blood pressure, diabetes, risk and protective factors for noncommunicable chronic diseases and reported morbidity, by sex. Vigitel. Brazilian capitals from 2006 to 2014.

		2006	2007	2008	2009	2010	2011	2012	2013	2014	APC (95%CI)
**Self-reported high blood pressure**											
	T	57.7	57.2	61.7	63.5	60.0	59.7	59.2	60.4	59.9	0.3 (−0.8;1.5)
	M	51.2	49.8	53.1	58.8	53.3	52.6	49.2	53.0	53.4	0.2 (−1.6;2.0)
	F	62.0	62.7	67.0	66.5	64.2	64.3	65.6	65.0	64.5	0.3 (−0.5;1.2)
**Self-reported diabetes**											
	T*	19.2	18.6	21.2	22.5	21.9	21.4	23.0	22.1	24.4	2.7 (1.3;4.1)
	M*	17.5	18.4	20.6	23.4	20.1	22.5	23.1	20.3	24.7	3.0 (0.9;5.2)
	F*	20.4	18.8	21.6	21.9	23.0	20.7	22.9	23.3	24.3	2.4 (1.3;3.5)
**Smokers**											
	T*	9.4	8.5	9.3	8.4	8.1	9.0	7.6	6.9	8.1	−2.8 (−4.1;−1.4)
	M*	13.2	12.2	13.8	12.2	10.3	14.0	11.1	10.1	10.4	−3.0 (-5.1;−0.9)
	F*	6.9	5.7	6.5	6.0	6.7	5.8	5.4	4.9	6.5	−2.2 (−4.3;−0.1)
**Abusive consumption of alcoholic beverages**											
	T	2.5	2.7	3.3	4.1	4.4	4.5	5.0	4.0	3.8	5.7 (−1.9;13.8)
	M	4.9	5.0	6.3	7.9	8.5	8.2	9.2	7.8	6.7	4.6 (-3.3;13.2)
	F	0.9	1.0	1.5	1.6	1.8	2.1	2.3	1.5	1.7	8.7 (−0.3;18.4)
**Overweight**											
	T*	52.4	50.8	53.6	54.2	56.6	54.3	58.5	56.3	57.8	1.5 (1.1;2.0)
	M*	50.6	50.3	51.5	55.3	58.1	51.3	57.4	54.6	57.3	1.5 (0.4;2.5)
	F*	53.6	51.3	54.9	53.5	55.6	56.4	59.1	57.4	58.3	1.6 (1.2; 2.0)
**Obesity**											
	T*	16.8	14.9	17.4	17.8	19.4	17.7	19	20.2	19.8	3.2 (2.0;4.3)
	M*	10.8	11.5	11.7	13.3	16.4	12.1	12.6	16.5	16.4	4.8 (1.5;8.2)
	F*	20.8	17.5	21.0	20.7	21.3	21.4	23.0	22.6	22.1	2.5 (1.5;3.5)
**Soft drink consumption**											
	T		17	11.2	13	12.9	14.1	12.1	11.4	10.1	−3.7 (-7.2;−0.1)
	M*		20.8	13.0	15.2	16.2	17.0	12.6	13.9	10.5	−4.9 (-9.1;−0.5)
	F		14.2	10.1	11.6	10.8	12.1	11.8	9.9	9.8	−2.5 (-5.6;0.6)
**Recommended consumption of fruits and vegetables**											
	T			26.3	25.0	22.2	29.9	28.4	26.8	27.8	2.2 (−1.5;6.0)
	M			22.7	19.8	16.8	27.4	25.5	22.7	22.7	2.8 (−4.1;10.2)
	F			28.5	28.3	25.6	31.6	30.2	29.4	31.4	2.0 (−0.2;4.3)
**Leisure-time physical activity**											
	T				22.6	20.7	22.5	23.6	22.3	22.8	1.2 (−0.9;3.3)
	M				27.1	23.8	27.4	31.1	26.2	24.7	−0.1 (−6.1;6.4)
	F				19.7	19.0	19.3	18.9	19.9	21.4	1.7 (−0.7;4.1)

* p≤0.05. T: total; M: male; F: female; APC: annual percent change; 95%CI: 95% confidence intervals.

**Table 2:** Interrupted time series of the prevalence of high blood pressure, diabetes, risk and protective factors for noncommunicable chronic diseases and reported morbidity, by sex. Vigitel. Brazilian capitals from 2015 to 2021.

		2015	2016	2017	2018	2019	2020	2021	APC (95%CI)
**Self-reported high blood pressure**									
	Total	59.6	64.2	60.9	60.9	59.3	60.6	61.0	−0.5 (−1.3;0.4)
	Male	56.1	57.9	55.8	56.7	55.5	55.9	57.1	−0.3 (−0.7;0.1)
	Female	61.9	67.8	64.2	63.6	61.6	63.9	63.7	−0.5 (−1.6;0.7)
**Self-reported diabetes**									
	Total	22.6	27.2	23.5	23.1	23.0	25.2	28.4	1.8 (−2.3;5.9)
	Male	24.2	25.9	23.0	24.6	24.6	24.6	28.7	1.3 (−0.9;3.6)
	Female	21.5	28.0	23.9	22.2	22.0	25.6	28.1	1.9 (-3.3;7.4)
**Smokers**									
	Total*	8.2	7.7	7.3	6.1	7.8	5.9	7.4	−3.4 (-5.5;−1.2)
	Male*	10.1	10.6	9.6	7.2	11.2	7.4	7.3	−5.4 (-9.5;−1.1)
	Female	6.9	6.0	5.8	5.4	5.7	4.8	7.5	−2.8 (-5.8;0.2)
**Abusive consumption of alcoholic beverages**									
	Total	3.7	4.6	3	4.1	4.1	5.7	5.8	7.7 (−0.2;16.2)
	Male	6.4	8.5	5.6	7.2	7.4	8.1	9.5	4.6 (−0.8;10.2)
	Female	2.0	2.3	1.4	2.0	2.1	4.0	3.3	11.1 (−2.1;26.0)
**Overweight**									
	Total*	57.3	57.7	59.6	60.6	59.8	60.9	60.7	1.0 (0.4;1.6)
	Male	57.5	55.2	56.8	61.1	60.6	58.5	60.7	1.3(−0.1;2.6)
	Female*	57.2	59.1	61.4	60.2	59.3	62.6	60.7	0.9 (0.1;1.8)
**Obesity**									
	Total	19.4	20.3	20.3	21.5	20.9	20.2	21.8	1.2 (−0.04;2.4)
	Male	15.5	15.1	15.4	18.3	18.0	15.0	16.8	1.4 (−2.6;5.5)
	Female*	21.9	23.4	23.4	23.6	22.7	23.7	25.3	1.4 (0.03;2.9)
**Soft drink consumption**									
	Total	9.4	9.9	7.8	7.0	8.4	10	8.7	−0.7 (-7.7;7.0)
	Male	11.6	9.7	10.7	8.0	10.8	12.2	11.2	1.5 (−4.7;8.1)
	Female	7.9	8.6	6.0	6.3	6.9	8.5	7.1	−0.9 (-7.8;6.6)
**Recommended consumption of fruits and vegetables**									
	Total	27.3	28.2	26.9	25.1	26.6	27.7	25.0	−1.0 (−2.7;0.6)
	Male	22.6	21.9	20.8	19.5	21.6	23.1	20.4	−0.6 (-3.5;2.4)
	Female	30.4	32.0	30.8	28.7	29.7	30.8	28.3	−1.2 (−2.5;0.2)
**Leisure-time physical activity**									
	Total	23.5	22.3	23.3	24.4	24.4	23.9	21.8	−0.4 (-3.0;2.2)
	Male	28.32	29.45	28.21	30.74	28.8	30.0	26.1	−0.2 (−1.5;1.0)
	Female	20.39	18.03	20.13	20.3	21.6	19.8	18.8	0.2 (−2.6;3.0)

* p≤0.05. APC: annual percent change; 95%CI: 95% confidence intervals.

Self-reported high blood pressure was stable in both periods. Diabetes increased significantly from 2006 to 2014 and stabilized from 2015 to 2021. Between 2006 and 2014, the prevalence of smokers decreased (p<0.05); and, from 2015 to 2021, the trends continued to decline among the total aged population and among males, however, there was stability in the prevalence for females (p=0.41). As for the abusive consumption of alcoholic beverages, the trends were stable in both periods (p>0.05). From 2015 to 2021, there was stability in the prevalence of overweight and obesity among aged males and for the total population in relation to obesity (p>0.05). Soft drinks consumption decreased in the first period (2007 to 2014) only among men, remaining stable among the other segments of the studied population. The consumption of fruits and vegetables and the practice of LTPA showed a trend of stability in both analyzed periods (Tables 1 and 2).

## DISCUSSION

Brazilian capitals registered, from 2006 to 2021: stability of high blood pressure for the total aged population; increased diabetes; reduction in the prevalence of smokers; increase in alcohol abuse, overweight, and obesity; decline in soft drink consumption; and stability of fruit and vegetable consumption and LTPA. Analyzing the interrupted time series, it is noteworthy that, between 2015 and 2021, there was stability in the prevalence of female smokers, overweight among men, obesity in the total aged population and among men, soft drinks consumption, and diabetes.

Increased burden of high blood pressure and diabetes is also driven by growth in modifiable risk factors, socioeconomic inequalities, and population aging^22,23^.

Since 1990, the prevalence of type 2 diabetes has increased by 30% in men and 26% in women. It is estimated that, by 2040, diabetes will be the third leading cause of death in Brazil^24^. A study with data from the National Health Survey (*Pesquisa Nacional de Saúde* – PNS), editions of 2013 and 2019, showed an increase in self-reported diabetes, from 6.2% (95%CI 5.9–6.8) to 7.7% (95%CI 7.4–8.0), and also high blood pressure, from 21.4% (95%CI 20.8–22.0) to 23.9% (95%CI 23.5–24.4)^25^. Socioeconomic inequalities related to NCDs should also be considered, since the highest incidence of these diseases occurs in black or brown individuals, illiterate or with incomplete primary education, who do not have private health insurance, and with lower income^26^. It is noteworthy that there has been a significant increase in the diagnosis, treatment, and control of these diseases, but there is still a need to reduce socioeconomic and health inequalities.

The prevalence of aged smokers has decreased over the years. However, among women, the decline occurred only in the first period analyzed (2006 to 2014). The reduction of smoking in Brazil is also observed among the adult population^27^, reflecting the commitment that the country assumed globally to reduce the prevalence of smokers by 30%^6^. Despite this reduction, the habit of smoking is still a Public Health problem, especially among the aged population, who, due to prolonged exposure to tobacco, are at greater risk of diseases, comorbidities, and premature deaths caused by tobacco^28^. Therefore, it is necessary to strengthen the regulatory role of the government, prevention measures, and the treatment of smokers^29^.

From 2006 to 2021, alcohol abuse increased among the total aged population and among women. This growth may be related to widowhood, loneliness, loss of friends, retirement, isolation, anxiety, depression, and stress^30^. It is important to highlight that the aged population is more vulnerable to the effects of alcohol and that, therefore, its consumption can cause cognitive and intellectual deficits, behavioral impairments, greater risk of exposure to falls and other injuries, in addition to medication interaction^31^. These effects are responsible for the increase in hospitalizations, deaths, and health costs^31^.

The prevalence of overweight and obesity has increased globally^32^, which can be attributed to behavioral, environmental, socioeconomic, genetic^33^, and changes in body composition that occur with aging^34^. Added to this is the spread of obesogenic environments and the increased consumption of ultra-processed and high-fat foods, a behavior that tends to increase in times of crisis, since such foods are more accessible when compared to fresh ones^35,36^. Obesity among aged people impacts not only morbidity and mortality, but also quality of life, increasing the risk of nursing home stays. Therefore, health promotion actions, such as healthy eating and the practice of physical activity, are effective and contribute to weight reduction, as well as the improvement of physical function and quality of life^33,37^. Efforts to halt the growth of overweight and obesity must be sustainable by the government and, above all, advance in regulatory measures, for taxation on sugary drinks and ultra-processed foods^38^.

In this context, a decline in soft drinks consumption was observed, however, considering the interrupted series, there was stability. Sugary drinks, such as soft drink, are the main sources of added sugar in the diet, and high consumption is considered an important risk factor for NCDs. Therefore, WHO recommends reducing sugar consumption to less than 10% of daily calorie intake and implementing fiscal policies to increase the price of sugary drinks^39^. Taxes were implemented in several countries and had a positive impact on reducing purchases or sales of these beverages^40^. These results are noteworthy, as no regulatory measure regarding food taxation has been implemented in Brazil, making it necessary to review this agenda^10^.

Fruits and vegetables contain essential nutrients for health and are essential for a healthy eating pattern. Regular consumption of these foods is characterized as a protective factor for NCDs, promotes healthy aging, is predictive of longevity, and brings cognitive benefits^41^. However, in the present study, no increase in consumption was observed among the aged, which is worrying. The low consumption of fruits and vegetables among the aged has been associated with economic conditions, rising food prices, lack of nutritional knowledge, reduced mobility and difficult access due to geographic location^42,43^. The increase in the consumption of fruits and vegetables among the aged population requires intersectionality, subsidy for access to healthy foods, market regulation strategies that allow the production and distribution of fruits and vegetables, policies to face food and nutritional insecurity in Brazil, in addition to macroeconomic and social policies that make it possible to reduce prices and increase family income^44,45^. Furthermore, it is necessary to encourage opportunities for communities and cities to implement actions that promote active, healthy, and sustainable aging, especially to deal with the vulnerabilities present in this population^43^.

The results of this study showed stability of LTPA in all analyzed periods, which highlights the need to increase it. There are factors that hinder the practice of physical activity among the aged population, such as: interpersonal barriers related to income, pain, illness or injury, physical limitation, fear of falling and getting hurt, and environmental barriers, such as lack of security, environmental characteristics, weather conditions, and the lack of places to practice physical activity close to home^46^. Thus, it becomes necessary to plan actions aimed at the aged public, to increase the practice of PSFT, seeking behavioral changes for the adoption of an active lifestyle, in addition to creating and maintaining environments that allow equitable access to places and spaces for the practice of physical activity in their cities and communities.

Analyzing the interrupted time series, from 2015 onward there was stability in the prevalence of some indicators analyzed in the present study, which may be a consequence of the economic crisis and austerity in Brazil, which contributed to the increase in unemployment and inequalities and to the reduction of investments in social and health promotion policies, culminating in the weakening of the government's regulatory role, in addition to directly affecting health services^11,47,48^.

This scenario became even more serious with the COVID-19 pandemic, which changed the behavioral patterns and lifestyles of the population^49^. Among the aged, the pandemic intensified issues that were already present, such as the loss of social support, the trauma of stigma, and isolation. There was also a reduction in access to health services, which may reduce the possibilities of diagnosing NCDs, in addition to the negative impact on daily activities, mental health, and cognition of the aged^50^. A study carried out with aged Brazilians who participated in the Behavioral Survey (ConVid) showed that, during the pandemic, only 8.3% of them (95%CI 6.4–10.7) continued to work normally, there was a decrease in income in almost half of the households of the aged, and 21.9% (95%CI 18.7–25.4) reported a worsening of their health status^51^. The pandemic also increased social and economic vulnerability, contributing to rising food prices and unemployment, consequently reducing access to food and increasing food insecurity and hunger^52,53^.

In the context of economic crisis, austerity, and pandemic, which compromise health results and indicators, it is paramount to integrate intersectoral public policies, strengthen actions to promote health, and encourage healthy living habits throughout life, as exposure to health risk factors in childhood and adolescence increases the chances of developing NCDs in adult life. It is also necessary to advance in regulatory measures and in the sustainability of programs and public policies to face NCDs and their risk factors in Brazil, as well as investing in social policies to reduce inequalities. In addition, it is necessary to expand access to diagnosis and treatment, in addition to improving the quality of care.

This study had the following limitations: collection by telephone, which may result in the exclusion of certain populations, though this issue is minimized by the use of data weighting factors, a measure that seeks to match the demographic characteristics of the Vigitel sample to the characteristics of the population total. Also, the age range considered (from 65 years of age), despite being restrictive, is the one that best fits when using the post-stratification weight. The fact that data were collected in a self-reported manner may result in under- or over-estimation of actual prevalence rates and generate less accurate estimates. However, validation studies of the Vigitel questionnaire show satisfactory reproducibility and validity results^54–56^.

In summary, it was found that, over the years, there has been an increase in diabetes, alcohol abuse, overweight and obesity, as well as a reduction in the prevalence of smokers and soft drink consumption, in addition to stability of the consumption of fruits and vegetables, the LTPA, and the occurrence of high blood pressure. Between 2015 and 2021, the prevalence of female smokers, overweight among men, obesity in the total aged population and among men, consumption of soft drinks and diabetes stabilized.

It is recommended to continue monitoring, to verify the trend of these indicators, mainly due to the economic context and the COVID-19 pandemic, in addition to the development of representative studies of the Brazilian aged population on the impact of the pandemic on morbidity and risk factors and of protection for NCDs.
