# Supplementary material for: Temporal trends of morbidities, and risk and protective factors for noncommunicable diseases in elderly residents in Brazilian capitals
Source: Rev Bras Epidemiol. 2023 Apr 21;26(Suppl 1):e230009. doi: 10.1590/1980-549720230009.supl.1 (PMC10176736; doi:10.1590/1980-549720230009.supl.1)
Supplement: Translation

# Translation: Tendências temporais de morbidades e fatores de risco e de proteção para doenças crônicas não transmissíveis em pessoas idosas residentes nas capitais brasileiras

**DOI:** 10.1590/1980-549720230009.supl.1.1

**Authors:** Alanna Gomes da Silva, Fabiana Martins Dias de Andrade, Edmar Geraldo Ribeiro, Deborah Carvalho Malta

**Affiliations:** I Universidade Federal de Minas Gerais, Escola de Enfermagem, Programa de Pós-Graduação em Enfermagem – Belo Horizonte (MG), Brasil.; II Universidade Federal de Minas Gerais, Faculdade de Medicina, Programa de Pós-Graduação em Saúde Pública – Belo Horizonte (MG), Brasil.

**Keywords:** Saúde do idoso, Doenças não transmissíveis, Fatores de risco, Fatores de proteção

## Abstract

**Objetivo:**

Analisar as tendências temporais das prevalências de morbidades e dos fatores de risco e de proteção para as doenças crônicas não transmissíveis em pessoas idosas residentes nas capitais brasileiras entre 2006 e 2021.

**Métodos:**

Estudo de série temporal com dados do Sistema de Vigilância de Fatores de Risco e Proteção para Doenças Crônicas por Inquérito Telefônico. Analisaram-se as variáveis: hipertensão arterial; diabetes; tabagismo; excesso de peso; obesidade; consumo de bebidas alcoólicas, refrigerantes, frutas e hortaliças; e prática de atividade física. Empregaram-se o modelo de regressão de Prais-Winsten e a séries temporais interrompidas (de 2006 a 2014 e de 2015 a 2021).

**Resultados:**

De 2006 a 2021, para a população total de idosos, houve aumento de diabetes (19,2 para 28,4%), do consumo de álcool (2,5 para 3,2%), do dexcesso de peso (52,4 para 60,7%) e da obesidade (16,8 para 21,8%), e redução do tabagismo (9,4 para 7,4%) e consumo de refrigerantes (17,0 para 8,7%). Pelas séries interrompidas, entre 2015 e 2021, houve estabilidade da prevalência de diabetes, fumantes do sexo feminino, excesso de peso nos homens, obesidade na população total e no sexo masculino e consumo de refrigerante.

**Conclusão:**

Ao longo dos anos houve mudanças e piora dos indicadores analisados, como aumento de diabetes, do consumo de álcool, do excesso de peso e da obesidade, o que reforça a importância do monitoramento contínuo e da sustentabilidade de programas de promoção da saúde, especialmente no contexto de crise econômica, austeridade e pandemia decorrente da COVID-19.

## INTRODUÇÃO

O Brasil está em um processo de transição demográfica e epidemiológica decorrente da diminuição das taxas de fecundidade e natalidade, bem como do aumento da expectativa de vida, da redução das doenças infecciosas e do crescimento de doenças crônicas não transmissíveis (DCNT)^1^.

O envelhecimento populacional é considerado um dos principais ganhos da sociedade, pois reflete os avanços sociais, tecnológicos e de saúde^2^. Estimativas da Organização das Nações Unidas mostram que a população global com 65 anos ou mais deverá aumentar de 10%, em 2022, para 16%, em 2050^3^. No Brasil, as projeções do Instituto Brasileiro de Geografia e Estatística (IBGE) demonstram que a população idosa será de 18,6%, em 2030, e de 33,7%, em 2060^4^.

As DCNT são as mais frequentes entre a população idosa, sendo também as principais causas de morte e de incapacidades^5^. Os quatro principais grupos de DCNT (cardiovasculares, câncer, doenças respiratórias crônicas e diabetes) apresentam fatores de risco modificáveis e em comum, como tabagismo, consumo abusivo de bebida alcoólica, inatividade física e alimentação inadequada, os quais acarretam inúmeras consequências sociais e econômicas para os indivíduos, as famílias e a sociedade^6^. Em contrapartida, a adoção de hábitos saudáveis, como a prática de atividade física e o consumo de frutas e hortaliças, pode reduzir o risco de desenvolvimento dessas doenças, além de auxiliar no controle do peso e melhorar a qualidade de vida e a saúde mental^7^.

Em função da gravidade das DCNT e de seu impacto para a Saúde Pública, esforços nacionais foram feitos para implementar políticas, programas e ações para a prevenção e o controle dessas doenças e de seus fatores de risco, além do cuidado integral e tratamento^6,7^. Há também as políticas e diretrizes que propõem ações, serviços e programas dirigidos à atenção integral à saúde das pessoas idosas^8,9^. Contudo, a partir de meados de 2014, ocorreram crises econômicas e governamentais no Brasil, com a implementação de políticas de austeridade que contribuíram para gerar desemprego, aumentar as desigualdades e afetar negativamente o sistema de saúde e de proteção social^10,11^. Soma-se a isso a pandemia de COVID-19, que contribuiu para redução do acesso aos serviços de saúde e piora das condições de vida, principalmente entre os idosos com DCNT^12,13^.

No contexto nacional de envelhecimento populacional e aumento das DCNT, acompanhado do cenário de crise, austeridade e pandemia de COVID-19, poucos estudos brasileiros foram feitos, especialmente com análises de séries históricas. Além disso, torna-se importante o acompanhamento dos indicadores de saúde da população idosa, a fim de verificar as mudanças ocorridas ao longo dos anos e conhecer a realidade em que estão inseridos e os seus hábitos de vida. Assim, espera-se contribuir com a vigilância, o controle e a prevenção das DCNT, para que os idosos brasileiros alcancem a longevidade com qualidade de vida.

Este artigo teve o objetivo de analisar as tendências temporais das prevalências de morbidades e dos fatores de risco e de proteção para as DCNT em pessoas idosas residentes nas capitais brasileiras entre 2006 e 2021.

## MÉTODOS

### Desenho do estudo e fonte dos dados

Trata-se de estudo de série temporal, no período de 2006 a 2021, com dados do Sistema de Vigilância de Fatores de Risco e Proteção para Doenças Crônicas por Inquérito Telefônico (Vigitel).

O Vigitel é um inquérito telefônico de base populacional que monitora anualmente, desde 2006, a frequência e a distribuição dos principais fatores de risco e de proteção para as DCNT^14^. Os procedimentos de amostragem empregados visam obter a representatividade das capitais dos 26 estados brasileiros e do Distrito Federal, por meio de amostras probabilísticas da população de adultos (≥18 anos de idade) que residem em domicílios com, pelo menos, uma linha telefônica fixa^14,15^.

### Amostra

Considerou-se, para este estudo, a população idosa com 65 anos ou mais de idade, por ser a mesma utilizada para a pós-estratificação nas análises do Vigitel.

Entre 2006 e 2022, entrevistaram-se aproximadamente 162.673 pessoas idosas com 65 anos ou mais, sendo: 6.069 (2006); 6.215 (2007); 6.679 (2008); 6.886; (2009); 7.340 (2010); 8.074 (2011); 7.580 (2012); 10.400 (2013); 8.417 (2014); 13.349 (2015); 13. 649 (2016); 15.727 (2017); 15.338 (2018); 17.583 (2019); 8.941 (2020); e 10.426 (2021).

### Variáveis

Para o presente estudo, incluíram-se as seguintes variáveis avaliadas pelo Vigitel:

- Morbidade: hipertensão arterial e diabetes autorreferida.
- Fatores de risco: tabagismo; consumo abusivo de bebidas alcoólicas; excesso de peso; obesidade; e consumo de refrigerantes (variável acrescentada ao Vigitel em 2007).
- Fatores de proteção: consumo recomendado de frutas e hortaliças (variável acrescentada ao Vigitel em 2008) e prática de atividade física no tempo livre — AFTL (variável acrescentada ao Vigitel em 2009).

A descrição completa das variáveis está no Material suplementar 1.

### Análises dos dados

As variáveis foram descritas em prevalência por ano de realização do inquérito e estratificadas segundo população total de idosos e sexo (feminino e masculino).

Este estudo utilizou a análise de séries temporais interrompidas, metodologia que tem como objetivo verificar se houve efeito de intervenção/evento/acontecimento nas tendências de determinada medida de interesse^16^. Assim, consideraram-se para a análise os seguintes períodos e os respectivos acontecimentos: 2006 a 2021 (período completo); 2006 a 2014 (implementação do plano de ações estratégicas para o enfrentamento das DCNT no Brasil 2011–2022 e estabilidade econômica)^6,11^; e 2015 a 2021 (ocorrência de crise econômica, com implementação de políticas de austeridade^17,18^, e pandemia de COVID-19, reconhecida pela Organização Mundial de Saúde [OMS], em 11 de março de 2020)^19^.

A análise das tendências foi realizada pela regressão linear generalizada de Prais-Winsten, que corrige o efeito da autocorrelação serial de primeira ordem^20^.

Realizou-se, também, o cálculo da variação percentual anual (*annual percent change* — APC) para cada variável analisada, a partir da seguinte fórmula^21^:


APC=(−1+10^β1)×100%

O beta 1 (β1) refere-se ao coeficiente angular da regressão de Prais-Winsten.

Calcularam-se, ainda, os intervalos de confiança de 95% (IC95%) das medidas de APC, utilizando-se a seguinte fórmula^21^:


IC95% mínimo=(−1+10^[β1−t×e])×100%

IC95% máximo=(−1+10^[β1+t×e])×100%

Em que: o *t* da fórmula refere-se ao teste *t* de Student pelos graus de liberdade para os períodos temporais e com nível de confiança de 95%, enquanto *e* corresponde ao erro padrão. Os valores de β1 da regressão de Prais-Winsten e do erro padrão foram gerados por programa de análise estatística.

Os resultados da regressão foram interpretados da seguinte forma: tendência significativa quando o β da regressão foi diferente de zero e o valor-p foi inferior ou igual a 0,05. Dessa forma, verificou-se tendência crescente quando o β foi positivo, de redução se o β foi negativo e estacionária quando não foi identificada diferença estatisticamente significativa.

Para análise dos dados, consideraram-se os pesos pós-estratificação usados no Vigitel^14,15^.

As análises foram realizadas no *software* Stata (Stata Corp. LP, College Station, Texas, United States), versão 14.0, e a organização dos dados e elaboração dos gráficos no Microsoft^®^ Office Excel 2016.

### Aspectos éticos

O Vigitel atendeu à Resolução n^o^ 466/2012, que envolve pesquisa com seres humanos. Os dados estão disponíveis para acesso e uso público, sendo sua coleta aprovada pela Comissão Nacional de Ética em Pesquisa em Seres Humanos do Ministério da Saúde. O termo de consentimento livre e esclarecido foi obtido oralmente no momento do contato telefônico com os entrevistados.

## RESULTADOS

A Figura 1 representa a série temporal das prevalências de hipertensão arterial e diabetes bem como dos fatores de risco e de proteção para as DCNT, de 2006 a 2021, segundo sexo, com a representação da interrupção, o que possibilita visualizar variações das prevalências dos indicadores a partir de 2015. Os resultados completos podem ser consultados no Material suplementar 2.

**Figura 1:** Séries temporais interrompidas das prevalências de hipertensão arterial, diabetes, dos fatores de risco e de proteção para as doenças crônicas não transmissíveis. Vigitel. Capitais brasileiras de 2006 a 2021.

A hipertensão arterial autorreferida aumentou de forma significativa entre os idosos do sexo masculino (APC=0,66%; IC95% 0,06–1,27). O diabetes apresentou tendência crescente para população total de idosos (APC=1,88; IC95% 1,13–2,64), homens (APC=2,32; IC95% 1,56–3,10) e mulheres (APC=1,60; IC95% 0,77–2,44) (Figura 1).

Em relação aos fatores de risco, considerando-se todo o período, a tendência foi de declínio das prevalências de tabagismo para o total de idosos (APC=−2,16; IC95% −2,77; −1,54) e para aqueles do sexo masculino (APC=-3,50; IC95% −4,38; −2,62). Em contrapartida, entre o sexo feminino a tendência foi de estabilidade. O consumo abusivo de bebidas alcoólicas apresentou tendência crescente entre a população total de idosos (APC=3,16; IC95% −0,09; 6,51) e as mulheres (APC=6,33; IC95% 2,65–10,15), com estabilidade entre os homens. Em relação ao excesso de peso, a tendência foi de aumento para o total de idosos (APC=1,11; IC95% 0,92–1,30), entre os homens (APC=1,13; IC95% 0,77–1,50) e entre as mulheres (APC=1,07; IC95% 0,85–1,29). De forma semelhante, a obesidade também cresceu de forma significativa, respectivamente: APC=1,87 (IC95% 1,29–2,45), APC=2,84 (IC95% 1,51–4,18) e APC=1,47 (IC95% 1,03–1,92). Para o consumo de refrigerante, a tendência foi decrescente para população total de idosos (APC=−4,32; IC95% −6,25; −2,35), entre os participantes do sexo masculino (APC=-3,99; IC95% −6,08; −1,86) e entre as mulheres (APC=−4,73; IC95% −6,74; −2,68) (Figura 1).

Quanto aos fatores de proteção para as DCNT, o consumo de frutas e hortaliças e a prática de AFTL permaneceram estáveis para a população total e para ambos os sexos (Figura 1).

As Tabelas 1 e 2 mostram os resultados das séries temporais interrompidas, realizadas em dois períodos (2006 a 2014 e 2015 a 2021), respectivamente, segundo sexo.

**Tabela 1:** Séries temporais interrompidas das prevalências hipertensão arterial, diabetes, dos fatores de risco e de proteção para as doenças crônicas não transmissíveis e morbidade referida, segundo sexo. Vigitel. Capitais brasileiras de 2006 a 2014.

		2006	2007	2008	2009	2010	2011	2012	2013	2014	APC (IC95%)
**Hipertensão arterial autorreferida**											
	T	57,7	57,2	61,7	63,5	60,0	59,7	59,2	60,4	59,9	0,3 (−0,8;1,5)
	M	51,2	49,8	53,1	58,8	53,3	52,6	49,2	53,0	53,4	0,2 (−1,6;2,0)
	F	62,0	62,7	67,0	66,5	64,2	64,3	65,6	65,0	64,5	0,3 (−0,5;1,2)
**Diabetes autorreferido**											
	T*	19,2	18,6	21,2	22,5	21,9	21,4	23,0	22,1	24,4	2,7 (1,3;4,1)
	M*	17,5	18,4	20,6	23,4	20,1	22,5	23,1	20,3	24,7	3,0 (0,9;5,2)
	F*	20,4	18,8	21,6	21,9	23,0	20,7	22,9	23,3	24,3	2,4 (1,3;3,5)
Fumantes											
	T*	9,4	8,5	9,3	8,4	8,1	9,0	7,6	6,9	8,1	−2,8 (−4,1;−1,4)
	M*	13,2	12,2	13,8	12,2	10,3	14,0	11,1	10,1	10,4	−3,0 (-5,1;−0,9)
	F*	6,9	5,7	6,5	6,0	6,7	5,8	5,4	4,9	6,5	−2,2 (−4,3;−0,1)
**Consumo abusivo de bebidas alcoólicas**											
	T	2,5	2,7	3,3	4,1	4,4	4,5	5,0	4,0	3,8	5,7 (−1,9;13,8)
	M	4,9	5,0	6,3	7,9	8,5	8,2	9,2	7,8	6,7	4,6 (-3,3;13,2)
	F	0,9	1,0	1,5	1,6	1,8	2,1	2,3	1,5	1,7	8,7 (−0,3;18,4)
**Excesso de peso**											
	T*	52,4	50,8	53,6	54,2	56,6	54,3	58,5	56,3	57,8	1,5 (1,1;2,0)
	M*	50,6	50,3	51,5	55,3	58,1	51,3	57,4	54,6	57,3	1,5 (0,4;2,5)
	F*	53,6	51,3	54,9	53,5	55,6	56,4	59,1	57,4	58,3	1,6 (1,2; 2,0)
**Obesidade**											
	T*	16,8	14,9	17,4	17,8	19,4	17,7	19	20,2	19,8	3,2 (2,0;4,3)
	M*	10,8	11,5	11,7	13,3	16,4	12,1	12,6	16,5	16,4	4,8 (1,5;8,2)
	F*	20,8	17,5	21,0	20,7	21,3	21,4	23,0	22,6	22,1	2,5 (1,5;3,5)
**Consumo de refrigerante**											
	T		17	11,2	13	12,9	14,1	12,1	11,4	10,1	−3,7 (-7,2;−0,1)
	M*		20,8	13,0	15,2	16,2	17,0	12,6	13,9	10,5	−4,9 (-9,1;−0,5)
	F		14,2	10,1	11,6	10,8	12,1	11,8	9,9	9,8	−2,5 (-5,6;0,6)
**Consumo recomendado de frutas e hortaliças**											
	T			26,3	25,0	22,2	29,9	28,4	26,8	27,8	2,2 (−1,5;6,0)
	M			22,7	19,8	16,8	27,4	25,5	22,7	22,7	2,8 (−4,1;10,2)
	F			28,5	28,3	25,6	31,6	30,2	29,4	31,4	2,0 (−0,2;4,3)
**Prática atividade física no tempo livre**											
	T				22,6	20,7	22,5	23,6	22,3	22,8	1,2 (−0,9;3,3)
	M				27,1	23,8	27,4	31,1	26,2	24,7	−0,1 (−6,1;6,4)
	F				19,7	19,0	19,3	18,9	19,9	21,4	1,7 (−0,7;4,1)

* p≤0,05. T: total; M: masculino; F: feminino; APC: annual percent change; IC95%: intervalos de confiança de 95%.

**Tabela 2:** Séries temporais interrompidas das prevalências hipertensão arterial, diabetes, dos fatores de risco e de proteção para as doenças crônicas não transmissíveis e morbidade referida, segundo sexo. Vigitel. Capitais brasileiras de 2015 a 2021.

		2015	2016	2017	2018	2019	2020	2021	APC (IC95%)
**Hipertensão arterial autorreferida**									
	Total	59,6	64,2	60,9	60,9	59,3	60,6	61,0	−0,5 (−1,3;0,4)
	Masculino	56,1	57,9	55,8	56,7	55,5	55,9	57,1	−0,3 (−0,7;0,1)
	Feminino	61,9	67,8	64,2	63,6	61,6	63,9	63,7	−0,5 (−1,6;0,7)
**Diabetes autorreferido**									
	Total	22,6	27,2	23,5	23,1	23,0	25,2	28,4	1,8 (−2,3;5,9)
	Masculino	24,2	25,9	23,0	24,6	24,6	24,6	28,7	1,3 (−0,9;3,6)
	Feminino	21,5	28,0	23,9	22,2	22,0	25,6	28,1	1,9 (-3,3;7,4)
**Fumantes**									
	Total*	8,2	7,7	7,3	6,1	7,8	5,9	7,4	−3,4 (-5,5;−1,2)
	Masculino*	10,1	10,6	9,6	7,2	11,2	7,4	7,3	−5,4 (-9,5;−1,1)
	Feminino	6,9	6,0	5,8	5,4	5,7	4,8	7,5	−2,8 (-5,8;0,2)
**Consumo abusivo de bebidas alcoólicas**									
	Total	3,7	4,6	3	4,1	4,1	5,7	5,8	7,7 (−0,2;16,2)
	Masculino	6,4	8,5	5,6	7,2	7,4	8,1	9,5	4,6 (−0,8;10,2)
	Feminino	2,0	2,3	1,4	2,0	2,1	4,0	3,3	11,1 (−2,1;26,0)
**Excesso de peso**									
	Total*	57,3	57,7	59,6	60,6	59,8	60,9	60,7	1,0 (0,4;1,6)
	Masculino	57,5	55,2	56,8	61,1	60,6	58,5	60,7	1,3(−0,1;2,6)
	Feminino*	57,2	59,1	61,4	60,2	59,3	62,6	60,7	0,9 (0,1;1,8)
**Obesidade**									
	Total	19,4	20,3	20,3	21,5	20,9	20,2	21,8	1,2 (−0,04;2,4)
	Masculino	15,5	15,1	15,4	18,3	18,0	15,0	16,8	1,4 (−2,6;5,5)
	Feminino*	21,9	23,4	23,4	23,6	22,7	23,7	25,3	1,4 (0,03;2,9)
**Consumo de refrigerante**									
	Total	9,4	9,9	7,8	7,0	8,4	10	8,7	−0,7 (-7,7;7,0)
	Masculino	11,6	9,7	10,7	8,0	10,8	12,2	11,2	1,5 (−4,7;8,1)
	Feminino	7,9	8,6	6,0	6,3	6,9	8,5	7,1	−0,9 (-7,8;6,6)
**Consumo recomendado de frutas e hortaliças**									
	Total	27,3	28,2	26,9	25,1	26,6	27,7	25,0	−1,0 (−2,7;0,6)
	Masculino	22,6	21,9	20,8	19,5	21,6	23,1	20,4	−0,6 (-3,5;2,4)
	Feminino	30,4	32,0	30,8	28,7	29,7	30,8	28,3	−1,2 (−2,5;0,2)
**Prática atividade física no tempo livre**									
	Total	23,5	22,3	23,3	24,4	24,4	23,9	21,8	−0,4 (-3,0;2,2)
	Masculino	28,32	29,45	28,21	30,74	28,8	30,0	26,1	−0,2 (−1,5;1,0)
	Feminino	20,39	18,03	20,13	20,3	21,6	19,8	18,8	0,2 (−2,6;3,0)

* p≤0,05. APC: annual percent change; IC95%: intervalos de confiança de 95%.

A hipertensão arterial autorreferida apresentou estabilidade nos dois períodos. Já o diabetes aumentou de forma significativa de 2006 a 2014 e estabilizou de 2015 a 2021. Entre 2006 e 2014, a prevalência de fumantes reduziu (p<0,05); e, de 2015 a 2021, as tendências continuaram declinando entre a população total de idosos e entre os homens, contudo, houve estabilidade das prevalências para o sexo feminino (p=0,41). Quanto ao consumo abusivo de bebidas alcoólicas, as tendências foram de estabilidade em ambos os períodos (p>0,05). De 2015 a 2021, houve estabilidade da prevalência de excesso de peso e da obesidade entre os idosos do sexo masculino e para a população total em relação a obesidade (p>0,05). O consumo de refrigerante reduziu no primeiro período (2007 a 2014) apenas entre os homens, mantendo-se estável entre as demais parcelas da população estudada. O consumo de frutas e hortaliças e a prática de AFTL apresentaram tendência de estabilidade em ambos os períodos analisados (Tabelas 1 e 2).

## DISCUSSÃO

De 2006 a 2021, ocorreu, nas capitais brasileiras: estabilidade da hipertensão arterial para a população total de idosos; aumento do diabetes; redução da prevalência de fumantes; aumento do consumo abusivo de bebidas alcoólicas, do excesso de peso e da obesidade; declínio do consumo de refrigerantes; e estabilidade do consumo de frutas e hortaliças e da prática de AFTL. Analisando-se as séries temporais interrompidas, destaca-se que, entre 2015 e 2021, houve estabilidade das prevalências de fumantes do sexo feminino, do excesso de peso entre os homens, da obesidade na população total de idosos e entre os homens, do consumo de refrigerante e do diabetes.

O aumento da carga da hipertensão arterial e do diabetes também é impulsionado pelo crescimento dos fatores de risco modificáveis, pelas desigualdades socioeconômicas e pelo envelhecimento populacional^22,23^.

Desde 1990, a prevalência do diabetes tipo 2 aumentou 30% em homens e 26% em mulheres. Estima-se que, até 2040, o diabetes deverá ser a terceira principal causa de morte no Brasil^24^. Um estudo com dados da Pesquisa Nacional de Saúde (PNS), edições de 2013 e 2019, evidenciou aumento do diabetes autorreferido, passando de 6,2% (IC95% 5,9–6,8) para 7,7% (IC95% 7,4–8,0), e também da hipertensão arterial, de 21,4% (IC95% 20,8–22,0) para 23,9% (IC95% 23,5–24,4)^25^. Deve-se considerar também as desigualdades socioeconômicas relacionadas às DCNT, visto que a maior incidência dessas doenças ocorre em indivíduos pretos ou pardos, analfabetos ou com ensino fundamental incompleto, que não têm plano de saúde privado e com menor renda^26^. Destaca-se que houve um importante incremento no diagnóstico, tratamento e controle dessas doenças, porém ainda há necessidade de reduzir as desigualdades socioeconômicas e de saúde.

A prevalência de idosos fumantes reduziu ao longo dos anos. Contudo, entre as mulheres, o declínio ocorreu apenas no primeiro período analisado (2006 a 2014). A redução do tabagismo no Brasil também é observada entre a população adulta^27^, sendo reflexo do compromisso que o país assumiu globalmente de reduzir em 30% a prevalência de fumantes^6^. Apesar dessa redução, o hábito de fumar ainda é um problema de Saúde Pública, especialmente entre a população idosa, que, devido à exposição prolongada ao fumo, tem maior risco de apresentar doenças, comorbidades e mortes prematuras provocadas pelo tabaco^28^. Por isso, é preciso fortalecer o papel regulatório do governo, as medidas prevenção e o tratamento das pessoas tabagistas^29^.

De 2006 a 2021, o consumo abusivo de bebidas alcoólicas aumentou entre a população total de idosos e entre as mulheres. Esse crescimento pode estar relacionado com viuvez, solidão, perda de amigos, aposentadoria, isolamento, ansiedade, depressão e estresse^30^. Torna-se importante destacar que a população idosa é mais vulnerável aos efeitos do álcool e que, portanto, seu consumo pode causar déficits cognitivo e intelectual, prejuízos ao comportamento, maior risco de exposição a quedas e outras lesões, além de ocorrer interação com os medicamentos utilizados^31^. Esses efeitos são responsáveis pelo aumento de hospitalizações, óbitos e custos com saúde^31^.

As prevalências de excesso de peso e obesidade têm aumentado globalmente^32^, podendo ser atribuídas aos fatores comportamentais, ambientais, socioeconômicos, genéticos^33^ e às mudanças da composição corporal que ocorrem com o envelhecimento^34^. Somam-se a isso a disseminação dos ambientes obesogênicos e o aumento do consumo de ultraprocessados e ricos em gorduras, comportamento que tende a crescer em períodos de crise, visto que tais alimentos são mais acessíveis, quando comparados com os alimentos frescos^35,36^. A obesidade em idosos impacta não apenas a morbidade e a mortalidade, mas também a qualidade de vida, aumentando o risco de institucionalização. Portanto, ações de promoção da saúde, como a alimentação saudável e a prática de atividade física, são efetivas e contribuem para a redução do peso, bem como a melhora da função física e da qualidade de vida^33,37^. Os esforços para deter o crescimento do excesso de peso e a obesidade devem ter sustentabilidade governamental e, sobretudo, avançar nas medidas regulatórias, para taxação sobre bebidas açucaradas e alimentos ultraprocessados^38^.

Neste contexto, observou-se declínio do consumo de refrigerante, contudo, considerando-se a série interrompida, houve estabilidade. As bebidas açucaradas, como o refrigerante, são as principais fontes de adição de açúcares na dieta e o alto consumo é considerado um importante fator de risco para as DCNT. Portanto, a OMS recomenda a redução do consumo de açúcar para menos de 10% da ingestão diária de calorias e a implementação de políticas fiscais para aumentar o preço das bebidas açucaradas^39^. Impostos foram implementados em vários países e impactaram positivamente para a redução das compras ou vendas dessas bebidas^40^. Esses resultados chamam a atenção, pois nenhuma medida regulatória referente à taxação de alimentos foi implantada no Brasil, fazendo-se necessário rever esta pauta^10^.

As frutas e hortaliças contêm nutrientes fundamentais para a saúde e são indispensáveis para um padrão saudável de alimentação. O consumo regular desses alimentos se caracteriza como fator de proteção para as DCNT, promove o envelhecimento saudável, é preditivo de longevidade e traz benefícios cognitivos^41^. No entanto, no presente estudo não foi verificado aumento do consumo entre os idosos, o que se torna preocupante. O baixo consumo de frutas e hortaliças entre os idosos tem sido associado às condições econômicas, à elevação dos preços dos alimentos, à falta de conhecimento nutricional, à mobilidade reduzida e à dificuldade ao acesso decorrente da localização geográfica^42,43^. O aumento do consumo de frutas e hortaliças entre a população idosa exige intersetorialidade, subsídio para acesso aos alimentos saudáveis, estratégias de regulamentação do mercado que oportunizem a produção e distribuição de frutas e hortaliças, políticas de enfrentamento da insegurança alimentar e nutricional no Brasil, além de políticas macroeconômicas e sociais que possibilitem a diminuição do preço e o aumento da renda familiar^44,45^. Ademais, é preciso estimular oportunidades para as comunidades e cidades implementarem ações que promovam o envelhecimento ativo, saudável e sustentável, especialmente para lidar com as vulnerabilidades presentes nessa população^43^.

Os resultados deste estudo mostraram estabilidade da AFTL em todos os períodos analisados, o que evidencia a necessidade de aumentá-la. Existem fatores que dificultam a prática de atividade física entre a população idosa, tais como: barreiras interpessoais relacionadas a renda, dor, doença ou lesão, limitação física, medo de cair e se machucar e barreiras ambientais, como a falta de segurança, as características do ambiente, as condições climáticas e a falta de locais para a prática de atividade física nas proximidades do domicílio^46^. Dessa forma, torna-se necessário o planejamento das ações voltadas ao público idoso, para aumentar a prática de AFTL, buscando modificações de comportamentos para a adoção de um estilo de vida ativo, além de criar e manter ambientes que permitam o acesso equitativo a lugares e espaços para a prática de atividade física nas suas cidades e comunidades.

Analisando-se as séries temporais interrompidas, a partir de 2015 houve estabilidade das prevalências de alguns indicadores analisados no presente estudo, o que pode ser consequência da crise econômica e da austeridade no Brasil, que contribuíram para o aumento do desemprego e das desigualdades e para a redução dos investimentos em políticas sociais e de promoção da saúde, culminando no enfraquecimento do papel regulatório do governo, além de terem afetado diretamente os serviços de saúde^11,47,48^.

Esse cenário tornou-se ainda mais grave com a pandemia de COVID-19, que alterou os padrões comportamentais e os estilos de vida da população^49^. Entre as pessoas idosas, a pandemia intensificou questões já presentes, como a perda de suporte social, o trauma de estigma e o isolamento. Houve também redução do acesso aos serviços de saúde, o que pode diminuir as possibilidades de diagnóstico das DCNT, além do impacto negativo nas atividades diárias, na saúde mental e na cognição dos idosos^50^. Um estudo realizado com idosos brasileiros que participaram da Pesquisa de Comportamentos (ConVid) mostrou que, durante a pandemia, apenas 8,3% (IC95% 6,4–10,7) continuaram trabalhando normalmente, houve diminuição da renda em quase metade dos domicílios dos idosos e 21,9% (IC95% 18,7–25,4) relataram piora do seu estado de saúde^51^. A pandemia também ampliou a vulnerabilidade social e econômica, contribuindo para a elevação dos preços dos alimentos e para o aumento do desemprego, consequentemente reduzindo o acesso aos alimentos e aumentando a insegurança alimentar e a fome^52,53^.

No contexto de crise econômica, austeridade e pandemia, o qual compromete os resultados e os indicadores de saúde, torna-se urgente a integração de políticas públicas intersetoriais, o fortalecimento das ações de promoção da saúde e de incentivo a hábitos de vida saudável ao longo de todo o curso da vida, pois a exposição aos fatores de risco para a saúde na infância e na adolescência aumenta as chances de desenvolvimento das DCNT na vida adulta. Deve-se, também, avançar nas medidas regulatórias e na sustentabilidade de programas e políticas públicas para o enfrentamento das DCNT e dos seus fatores de risco no Brasil, bem como investir em políticas sociais para redução das desigualdades. Ademais, é preciso ampliar o acesso ao diagnóstico e ao tratamento, além de melhorar a qualidade do cuidado.

Este estudo teve como limitações: a coleta por telefone, que pode resultar na não inclusão de determinadas populações, porém essa questão é minimizada pelo uso de fatores de ponderação dos dados, medida que busca igualar as características demográficas da amostra do Vigitel às características da população total. Também, a faixa etária considerada (a partir dos 65 anos de idade), apesar de restritiva, é a que melhor se ajusta ao ser empregado o peso pós-estratificação. O fato de os dados terem sido coletados de forma autorreferida pode resultar na sub ou superestimação das prevalências reais e gerar estimativas menos precisas. Contudo, estudos de validação do questionário do Vigitel mostram resultados satisfatórios de reprodutibilidade e validade^54–56^.

Em síntese, verificou-se que, ao longo dos anos, houve aumento de diabetes, do consumo abusivo de bebidas alcoólicas, do excesso de peso e da obesidade, bem como redução da prevalência de fumantes e do consumo de refrigerantes, além de estabilidade do consumo de frutas e hortaliças, da prática de AFTL e da ocorrência de hipertensão arterial. Entre 2015 e 2021, estabilizaram-se as prevalências de fumantes do sexo feminino, do excesso de peso entre os homens, da obesidade na população total de idosos e entre os homens, do consumo de refrigerantes e do diabetes.

Recomenda-se a continuidade do monitoramento, para verificar a tendência desses indicadores, principalmente pelo contexto econômico e da pandemia de COVID-19, além do desenvolvimento de estudos representativos da população idosa brasileira sobre o impacto da pandemia na morbidade e nos fatores de risco e de proteção para as DCNT.
